# Genome-Wide Association Mapping of Seedling Biomass and Root Traits Under Different Water Conditions in Wheat

**DOI:** 10.3389/fgene.2021.663557

**Published:** 2021-04-12

**Authors:** Iza Fatima, Yutian Gao, Xiangru Xu, Jingjing Jin, Shuonan Duan, Wenchao Zhen, Chaojie Xie, Jun Ma

**Affiliations:** ^1^College of Agronomy and Biotechnology, China Agricultural University, Beijing, China; ^2^College of Agronomy, Hebei Agricultural University, Baoding, China

**Keywords:** wheat, seedling biomass, root traits, GWAS, osmotic stress

## Abstract

Drought is a major threat to global wheat production. In this study, an association panel containing 200 Chinese wheat germplasms was used for genome-wide association studies (GWASs) of genetic loci associated with eight root and seedling biomass traits under normal water and osmotic stress conditions. The following traits were investigated in wheat seedlings at the four-leaf stage: root length (RL), root number (RN), root fresh weight (RFW), root dry weight (RDW), shoot fresh weight (SFW), shoot dry weight (SDW), total fresh weight (TFW), and total dry weight (TDW). A total of 323 and 286 SNPs were detected under two water environments, respectively. Some of these SNPs were near known loci for root traits. Eleven SNPs on chromosomes 1B, 2B, 4B, and 2D had pleiotropic effects on multiple traits under different water conditions. Further analysis indicated that several genes located inside the 4 Mb LD block on each side of these 11 SNPs were known to be associated with plant growth and development and thus may be candidate genes for these loci. Results from this study increased our understanding of the genetic architecture of root and seedling biomass traits under different water conditions and will facilitate the development of varieties with better drought tolerance.

## Introduction

Wheat (*Triticum aestivum* L.) is a widely cultivated crop in the world that provides the main source of calories and protein in the human diet (Shewry, 2009). In many regions of the world, wheat production suffered significant losses due to drought stress (Trethowan and Pfeiffer, 2000). Drought stress can induce significant morphological and physiological changes in plants, including stomatal closure, reductions in photosynthesis and transpiration, shoot and root growth inhibition, antioxidant production, and changes in hormonal composition (Szegletes et al., 2000; Lawlor and Cornic, 2002; Zhu, 2002). The yield loss caused by drought can be up to 92% (Farooq et al., 2014). Due to climate change, the frequency and severity of drought stress will significantly increase in the future and pose a threat to the food security of the rapidly increasing world population (IPCC, 2014).

The root system is vital for plants to obtain water and nutrients from the soil. A positive correlation between root system architecture and agronomic traits was reported by Cane et al. (2014). During grain filling, every millimeter of water extracted from the soil increased wheat yield by 55 kg/ha (Manschadi et al., 2006; Kirkegaard et al., 2007; Christopher et al., 2013). The root system is also a key structure to respond to water stress conditions and maintain yield under drought stress because plants with deep root systems and large root biomass could extract more water from deeper soil layers (Boyer, 1996; Sharma and Carena, 2016; Wasaya et al., 2018). A study on durum wheat showed that compared with shallow root genotypes, deep root genotypes had 16 to 35% larger grains and can increase grain yield by 35% and thousand-grain weight by 9% in environments with limited moisture (El Hassouni et al., 2018). Furthermore, unlike the consistent reduction of shoot biomass and grain yield under drought stress, the responses of wheat root biomass to drought might be negative, positive, or no response depending on the genotype and environmental factors, which make it an ideal target trait for the improvement of drought tolerance (Wasaya et al., 2018).

To date, a large number of quantitative trait loci (QTL) related to various root traits including root dry weight, seminal root number, total root length, root diameter, number of root tips, root number, etc., have been reported in wheat (Bai et al., 2013; Liu et al., 2013, 2019; Ayalew et al., 2017; Xie et al., 2017; Alahmad et al., 2019; Beyer et al., 2019). Of these QTL, some QTL were specific to water stress conditions. For example, Ayalew et al. (2017) identified four root length QTL specific to drought stress conditions on chromosomes 1A, 3A, and 7B. Similarly, Liu et al. (2013) reported several QTL for maximum root length and seminal root area on chromosomes 1A, 2A, 5A, and 5D. Several QTL controlling plant height and shoot dry weight also affected various root traits such as root length and root biomass, indicating the important roles of the root system on plant growth and development (Cao et al., 2014; Iannucci et al., 2017).

In this study, we performed a genome-wide association study (GWAS) to identify sets of markers associated with root and seedling biomass traits in a panel of 200 Chinese wheat germplasms under normal water and PEG-induced osmotic environments via hydroponic culture. The results will increase our understanding of the genetic architecture of root and seedling biomass traits under different water conditions and will facilitate the development of varieties with better drought tolerance.

## Materials and Methods

### Plant Material

An association panel consisting of 200 Chinese varieties of wheat collected from different wheat production regions of China were used in this study (Supplementary Table 1). Most of them were from the Yellow and Huai River Valley, one of the major wheat-producing regions in China (Jin et al., 2020).

### Phenotypic Evaluation

Seeds were first selected by removing small and shriveled kernels. Seeds from each variety were soaked in 70% sodium hypochlorite solution for 10 min to sterilize and then washed two to three times with distilled water. Following that, seeds were germinated in petri dishes at room temperature. The seedlings with 0.5 cm length of coleoptiles were rolled in germination paper after 1–2 days. Correspondingly, seedlings were transferred 1 day later to a container with 1/2 Hoagland nutrient solution in a greenhouse with 60% humidity, 25°C temperature, and 10/14 h (day/night) timing using automatic timer. On the seventh day, eight seedlings of each variety were grown to 1/2 Hoagland solution with 20% polyethylene glycol (PEG) 6000 (Sinopharm Chemical Reagent Co. Ltd, China) for drought treatment, whereas the remaining eight seedlings were kept in 1/2 Hoagland solution for control. The solution containing PEG was changed every 3 days to keep the water potential stable. For both controlled and drought environments, the experiment was repeated three times.

When the seedlings were at the four-leaf stage, eight traits, including root length (RL), root number (RN), root fresh weight (RFW), root dry weight (RDW), shoot fresh weight (SFW), shoot dry weight (SDW), total fresh weight (TFW), and total dry weight (TDW), were evaluated under both normal and drought environments. The roots of the seedlings were first washed before measurement. The longest root among all the roots of a seedling was selected for the measurement of RL with the help of a measuring scale and was expressed in centimeters (cm). For the measurement of RFW, the excessive water was removed by pressing the roots gently with a tissue paper sheet. For the measurement of RDW, samples were dried to constant weight by incubating them in small paper bags at 80°C for 48 h. The SFW and SDW were measured in a similar way.

### Statistical Analysis

To analyze the variation among all eight traits, different statistical tools such as mean, median, sum, variance, range, standard error of the mean, confidence interval of the mean, standard deviation, and coefficient of variance were applied by using a statistical package “pastecs” in R software. The following formula was used for the calculation of broad sense heritability (*H*^2^) of various traits:

$$\begin{array}{l} {\text{H}}^{2}=V_{\text{G}}/\left(V_{\text{G}}+V_{\text{E}}\right) \end{array}$$

Here, V_G_ denotes genetic variance, and V_E_ represents environmental variance (Ehlers et al., 2009). R corrplot was employed to analyze the correlation of each trait. For each line, the lme4 package in R was used for the estimation of the best linear unbiased predictors (BLUP), which were later used for GWAS analysis. Each trait was analyzed within eight plants in each accession, and the mean of all eight traits was used for consecutive statistical analysis and GWAS.

### Genome-Wide Association Analysis and Prediction of Candidate Gene

Molecular marker data of the 200 germplasms in the association panel was extracted from our previous publication (Jin et al., 2020). The Genomic Association and Prediction Integrated Tool (GAPIT) package in R (Version 4.0.3.) was used for GWAS analysis (Lipka et al., 2012). GWAS was performed using the mixed linear model (PCA + K), and the variance–covariance kinship matrix (*K*) was calculated by the VanRaden method in R (VanRaden, 2008; Zhang et al., 2010; Lipka et al., 2012; Jin et al., 2020). A suggestive threshold of *P* value equal to 1.0E−3 (*P* = 1/n, *n* = effective SNP number) was used to estimate the significant SNPs (Sun et al., 2017; Jin et al., 2020). The CMplot package in R was used to draw Manhattan plots, which are showing the SNPs identified for root traits in GWAS using BLUP values of 200 wheat germplasm (LiLin-Yin, 2020).

The stable SNPs in the three experiments were selected for the favorable allele analysis. In the analysis of allele effects on each trait, alleles with positive effects leading to higher values of root traits were described as “favorable alleles,” whereas those with lower values were “unfavorable alleles” (Liu et al., 2019). For the investigation of potential candidate genes, the EnsemPlants database (http://plants.ensembl.org) was used to download the genes within 4 Mb LD block on both sides of the significant SNPs in both controlled and drought environments (Jin et al., 2020). Gene annotation, the relative homologous rice, and the *Arabidopsis* gene of particular wheat genes were also investigated through the Triticeae-Gene Tribe (TGT) website (http://wheat.cau.edu.cn/TGT/index.html) (Chen et al., 2020).

## Results

### Phenotypic Evaluation

All phenotypic traits in both controlled and drought environments showed continuous and significantly wide variations in 200 wheat germplasms. The basic statistics of these phenotypic traits is shown in Table 1. The mean values of RDW, SDW, TDW, RFW, SFW, TFW, RN, and RL were significantly higher in a controlled environment (0.014, 0.035, 0.049, 0.21, 0.469, 0.679, 6.061, and 27.332, respectively) compared with those in a drought environment (0.017, 0.022, 0.039, 0.107, 0.174, 0.282, 4.947, and 18.796, respectively) as shown in Table 1. The coefficient of variation for all eight traits in controlled and drought environments ranged from 2.1 to 14.6% and 1.7 to 10.8% respectively. The values of standard deviation (SD) ranged from 0.001 to 0.599 in the controlled environment and from 0.001 to 0.335 in the drought environment (Table 1).

**Table 1:** Basic statistics of root traits for 200 accessions grown in controlled and drought conditions.

**Environment**	**Trait**	**Min**	**Max**	**Ave**	**SD**	**Coef. of var (%)**	***H*^**2**^**
Drought	RL (cm)	17.806	19.650	18.796	0.335	1.7	0.517
	RN	3.815	5.708	4.947	0.313	6.3	0.793
	RFW (g)	0.088	0.125	0.107	0.006	6.4	0.544
	RDW (g)	0.013	0.020	0.017	0.001	7.0	0.650
	SFW (g)	0.141	0.222	0.174	0.015	9.0	0.654
	SDW (g)	0.017	0.031	0.022	0.002	10.8	0.766
	TFW (g)	0.231	0.345	0.282	0.019	7.0	0.628
	TDW (g)	0.031	0.049	0.039	0.003	8.3	0.731
Control	RL (cm)	25.635	28.815	27.332	0.599	2.1	0.271
	RN	4.685	7.551	6.061	0.514	8.4	0.692
	RFW (g)	0.155	0.323	0.210	0.026	12.4	0.619
	RDW (g)	0.010	0.022	0.014	0.001	12.5	0.572
	SFW (g)	0.348	0.654	0.469	0.052	11.2	0.668
	SDW (g)	0.010	0.056	0.035	0.005	14.6	0.676
	TFW (g)	0.505	0.969	0.679	0.078	11.5	0.667
	TDW (g)	0.035	0.076	0.049	0.006	13.8	0.661

*Trait abbreviations: RL, root length; RN, root number; RFW, root fresh weight; RDW, root dry weight; SFW, shoot fresh weight; SDW, shoot dry weight; TFW, total fresh weight; TDW, total dry weight; SD, standard deviation; H^2^, broad-sense heritability*.

**Figure 1:**
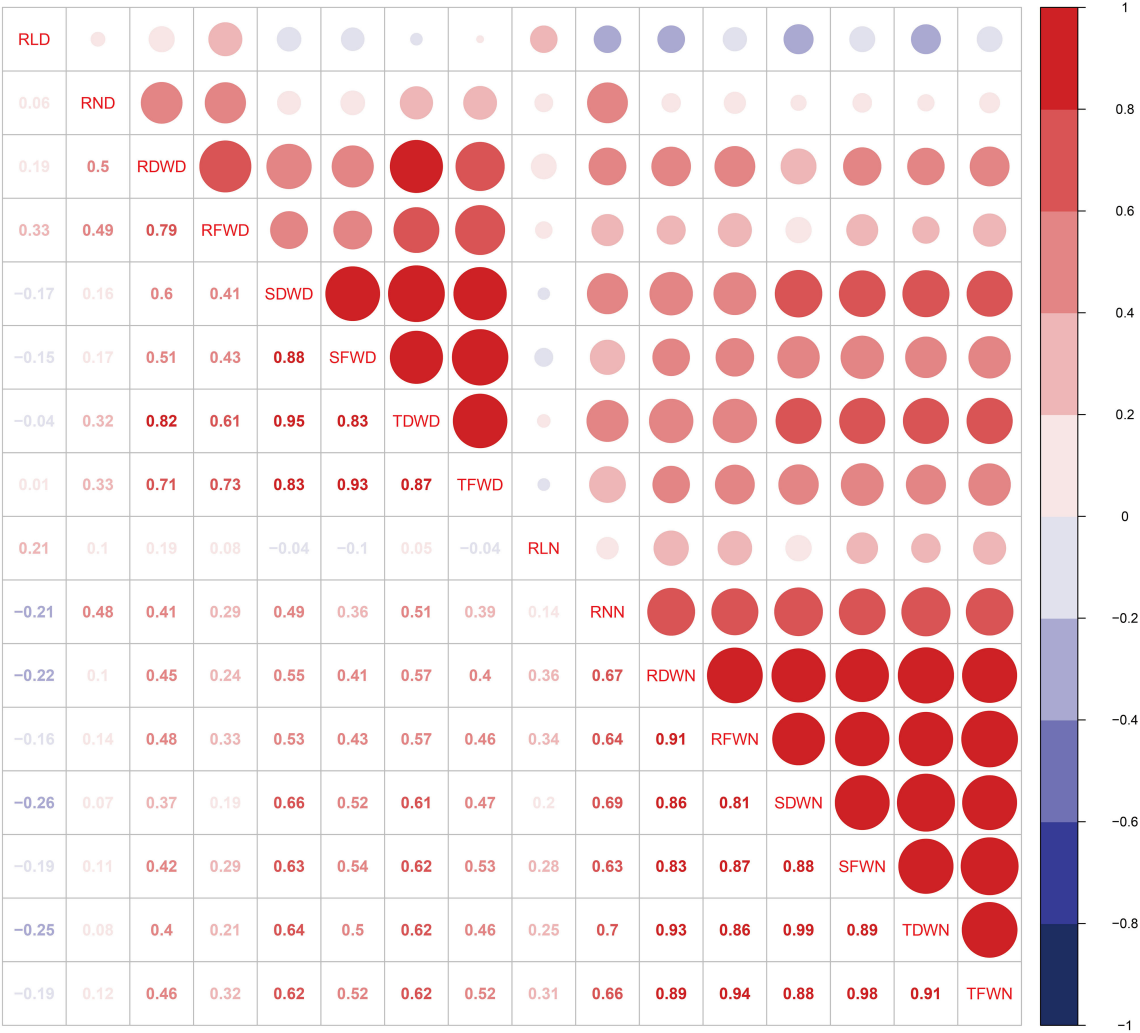
Correlation analyses between eight root and seedling biomass traits for each environment. Trait abbreviations: RLD, root length in drought environment; RLN, root length in controlled environment; RND, root number in drought environment; RNN, root number in controlled environment; RDWN, root dry weight in controlled environment; RDWD, root dry weight in dry environment; RFWN, root fresh weight in controlled environment; RFWD, root fresh weight in dry environment; SFWN, shoot fresh weight in controlled environment; SFWD, shoot fresh weight in dry environment; SDWN, shoot dry weight in controlled environment; SDWD, shoot dry weight in drought environment; TFWN, total fresh weight in controlled environment; TFWD, total fresh weight in drought environment; TDWN, total dry weight in controlled environment; TDWD, total dry weight in drought environment.

Correlation coefficients (*r*^2^) among different phenotypic traits were analyzed to quantify the relationship between all eight traits under both controlled and drought environments (Figure 1). A strong correlation was detected in various root traits under both environments. For example, TFWD is positively correlated with SFWD and TDWD with a value range of (0.83) and (0.87) in the drought environment, while RFWN was positively correlated with SDWN (0.81), SFWN (0.87), TDWN (0.86), and TFWN (0.94), respectively (Figure 1). Most of the investigated traits showed a strong broad sense heritability (*H*^2^), ranging from 0.271 (RL) to 0.692 (RN) and 0.517 (RL) to 0.793 (RN) in the controlled and drought environments, respectively (Table 1). However, the *H*^2^ of RL under controlled environments was relatively low (0.271), suggesting a significant environmental effect (Table 1).

### Marker–Trait Associations

Based on the BLUP values in the three experiments, a total of 609 SNPs were significantly associated with eight root and seedling biomass traits in the current study (Supplementary Table 2). The B genome has the highest number of SNPs (440), followed by the D (93) and A (65) genomes. These significant SNPs were distributed on 21 chromosomes except 2A, 4A, 5A, and 7A. From these SNPs, a total of 323 SNPs related to root and seedling biomass traits were identified in the normal water condition. Chromosome 2B had the largest proportion of SNPs (19.50%), followed by chromosome 5B. Additionally, 115 SNPs were associated with multiple traits, including 30 SNPs on chromosomes 5B (22), 4D (6), and 5D (2) associated with RDW, RFW, SDW, SFW, TDW, and TFW (Supplementary Table 2). A total of 286 SNPs related to root and seedling biomass traits were observed under the drought environment. These SNPs were distributed on all the 21 chromosomes of wheat except 6A (Supplementary Table 2). The same as those in the controlled environment, most of these SNPs were on chromosome 2B (38.11%) and chromosome 5B (26.57%).

**Figure 2:**
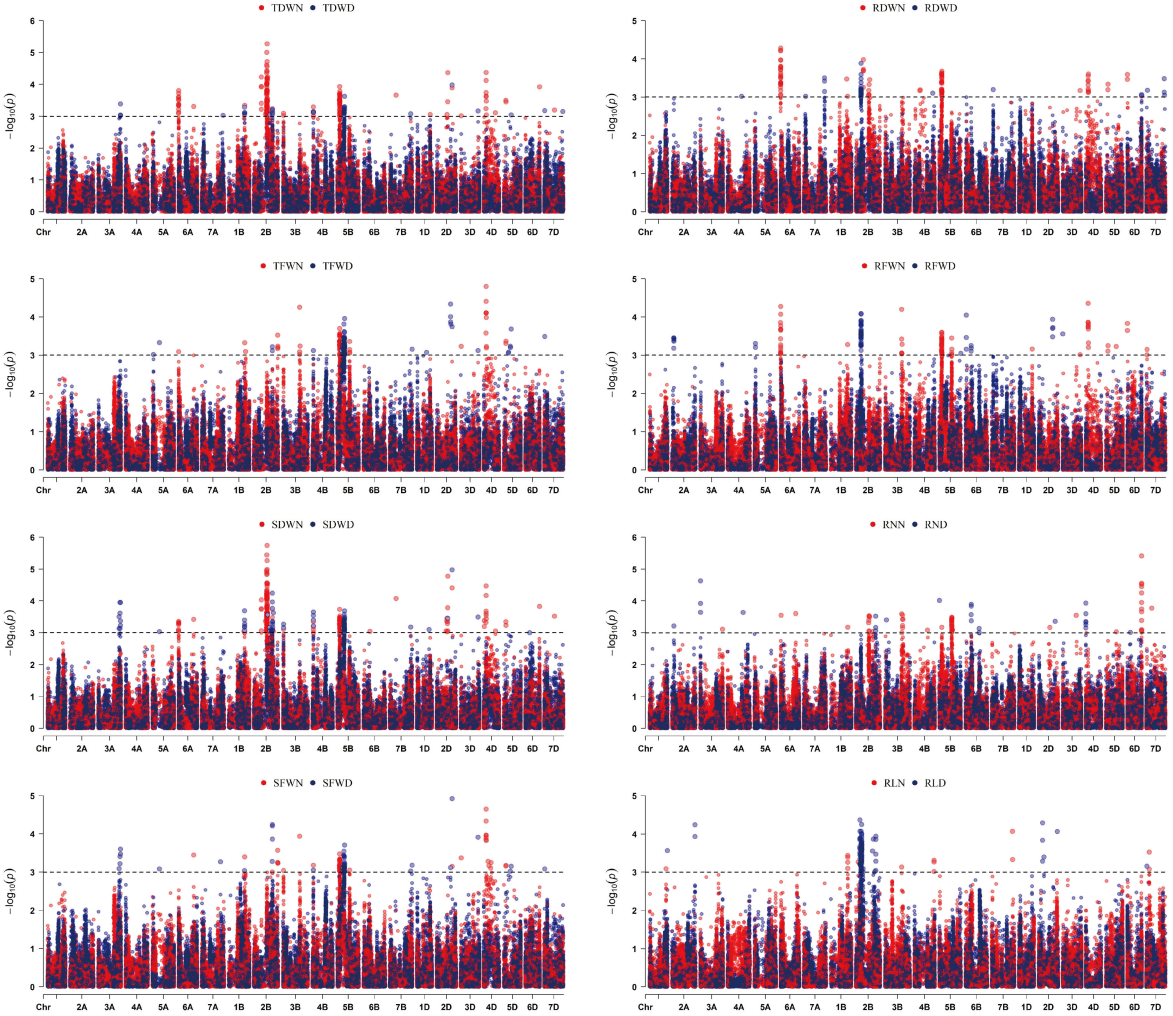
Manhattan plots for the eight root and seedling biomass traits identified by genome-wide association study (GWAS) using BLUP values. The dashed line represents the significance threshold (–log10 *P* = 3.0). Trait abbreviations: RLD, root length in drought environment; RLN, root length in controlled environment; RND, root number in drought environment; RNN, root number in controlled environment; RDWN, root dry weight in controlled environment; RDWD, root dry weight in dry environment; RFWN, root fresh weight in controlled environment; RFWD, root fresh weight in dry environment; SFWN, shoot fresh weight in controlled environment; SFWD, shoot fresh weight in dry environment; SDWN, shoot dry weight in controlled environment; SDWD, shoot dry weight in drought environment; TFWN, total fresh weight in controlled environment; TFWD, total fresh weight in drought environment; TDWN, total dry weight in controlled environment; TDWD, total dry weight in drought environment.

Moreover, statistical analysis identified 11 SNPs that appeared to be significantly associated (P < 0.001) with RN, RDW, SFW, SDW, TFW, and TDW under two water conditions (Table 2). These 11 loci were distributed on chromosomes 1B, 2B, 4B, and 2D, respectively. Two SNP loci (*Affx-111601113* on chromosome 2B and *Affx-88425798* on chromosome 2D) explained 14.5 to 30.6% of the phenotypic variance in RNN (Table 2, Figure 2). These two SNP loci also had significant effects on SDWN, TDWN, and SDWD. Similarly, one SNP (*Affx-109979441*) on chromosome 1B was associated with RDWN, SFWN, TFWN, TDWN, SDWD, and TDWD; five on chromosome 2B (*Affx-111601113, Affx-111326878, Affx-111434051, Affx-109782056*, and *Affx-92897136*) were associated with RNN, SFWN, SDWN, TDWN, SFWD, SDWD, TFWN, and TDWD; three on chromosome 4B (*Affx-109674658, Affx-88444969*, and *Affx-88596529*) showed pleiotropic effect for SDWN, TDWN, SDWD, and TDWD, respectively (Table 2).

**Table 2:** The SNP significantly associated with pleiotropic effect in different phenotyping traits in two environments.

**Marker^a^**	**Chromosome**	**Position^b^**	**Pleiotropic effect**	**SNP^c^**	***P* value**
Affx-109979441	1B	533184310	RDWN, SFWN, SDWN, TFWN, TDWN, SDWD, TDWD	C	<0.001
Affx-109782056	2B	607064966	SDWN, SFWD, SDWD, TDWN, TFWD, TDWD	C	<0.001
Affx-111326878	2B	606037476	SDWN, TDWN, SFWD, SDWD, TDWD	T	<0.001
Affx-111434051	2B	606938708	RDWN, SDWN, SFWD, SDWD, TFWD, TDWD	T	<0.001
Affx-111601113	2B	448398290	RNN, SDWN, TDWN, SDWD	A	<0.001
Affx-92897136	2B	641102486	SDWN, SDWD	C	<0.001
Affx-109617080	2D	514348182	RDWN, SDWN, TDWN, SFWD, SDWD, TFWD, TDWD	C	<0.001
Affx-88425798	2D	375362652	RNN, SDWN, TDWN, SDWD	C	<0.001
Affx-109674658	4B	37700884	SDWN, TDWN, SDWD, TDWD	T	<0.001
Affx-88444969	4B	37845625	TDWN, SDWD	G	<0.001
Affx-88596529	4B	37735225	SDWN, SDWD, TDWN	C	<0.001

*Trait abbreviations: RNN, root number in controlled environment; RDWN, root dry weight in controlled environment; SFWN, shoot fresh weight in controlled environment; SDWN, shoot dry weight in controlled environment; TFWN, total fresh weight in controlled environment; TDWN, total dry weight in controlled environment;, SFWD, shoot fresh weight in drought environment; SDWD, shoot dry weight in drought environment; TFWD, total fresh weight in drought environment; TDWD, total dry weight in drought environment*.

a *Representative marker at a specific locus*.

b *Physical position of SNP marker in base pair (bp)*.

c *Indication of favorable allele (SNP)*.

**Figure 3:**
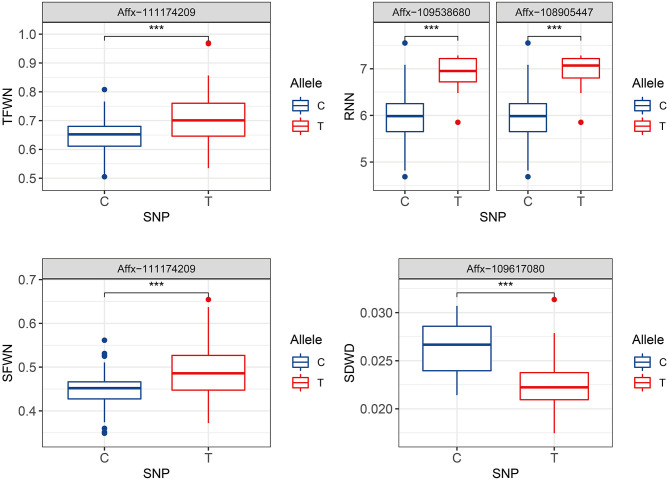
Comparison of the allele effects of four environmentally stable SNPs on chromosomes 2D, 4D, and 6D. Trait abbreviations: TFWN, total fresh weight in controlled environment; SFWN, shoot fresh weight in controlled environment; RNN, root number in controlled environment; SDWD, shoot dry weight in drought environment. ***p < 0.001.

Stability is an important parameter to evaluate a particular QTL. Of the SNPs identified by GWAS analyses based on BLUP value, four SNPs on chromosomes 2D, 4D, and 6D were stable in all of the three replicates (Supplementary Table 2). The favorable allele of SNP *Affx-111174209* on chromosome 4D led to an increase from 0.45 to 0.48 for SFWN, and from 0.65 to 0.71 for TFWN (Figure 3). The SNP (*Affx-109617080*) on chromosome 2D led to an increase from 0.0209 to 0.0254 for SDWD. Two SNPs on chromosome 6D including *Affx-109538680* and *Affx-108905447* led to an increase of 0.97 and 1.1 for RNN, respectively (Figure 3).

### Candidate Genes for the SNPs Stable Under Two Water Conditions

Based on genes annotated in Chinese spring reference genome, a total of 442 genes were identified in the 4 Mb (LD block) region on each side of 11 significant SNPs (Supplementary Table 3). Some of these genes raised our interests due to their reported roles in plant growth and development. These genes included *TraesCS1B02G310200* near *Affx*-*109979441* on chromosome 1B, which encoded the transcription repressor OFP4, *TraesCS2B02G423500* on chromosome 2B and *TraesCS2D02G402400* on chromosome 2D encoding rolling and erect leaf 2 protein, two genes on chromosome 4B including *TraesCS4B02G048400* encoding G-type lectin S-receptor-like serine/threonine protein kinase, and *TraesCS4B02G049700* encoding 26S proteasome non-ATPase regulatory subunit 8, respectively.

## Discussion

Due to the difficulty in phenotyping root traits, direct selection for variation in root characteristics is impractical (Reynolds et al., 2007). Molecular screens are likely to have a considerable cost-benefit advantage over the phenotyping method (Tuberosa and Salvi, 2006). In this study, a total of 164 SNPs were associated with root traits under controlled environment, whereas 152 SNPs were identified under osmotic stress (Supplementary Table 2). These SNPs are useful genetic resources for the improvement of root traits and drought tolerance in wheat. Some of these SNP loci are closely located to the known loci associated with root traits based on the reference genome of IWGSC V1.1. For example, under drought stress condition, three SNP loci including *Affx-88733278, Affx-109672297*, and *Affx-110800753* for root length on chromosome 2B are 2.29, 1.79, and 3.39 Mb away from *AX_111251784, AX_94405934*, and *AX_108756976*, which were associated with total root length in a previous study (Liu et al., 2019). Three SNPs (*Affx-110656000, Affx-111718859*, and *Affx-88565514*) on chromosome 3A for root number was about 0.7 Mb away from an SNP (*S7_12487861*) for branched root length (Beyer et al., 2019). Under normal water conditions, *Affx-110668350* on chromosome 6A for RDW and *Affx-109434039* on chromosome 7D for RL were 0.48 and 1 Mb away from *S16_7327093* and *S21_99959518* for root diameter, respectively (Beyer et al., 2019).

Similar to the SNP specific to a single water condition, several SNPs under two water conditions were closely located to known QTL for root traits or grain yield-related traits (Table 2). For example, the three SNP loci (*Affx*-*109782056, Affx*-*111326878*, and *Affx*-*111434051*) on chromosome 2B for seedling biomass traits were in the same chromosome region as a QTL (*QSRN.cgb-2B*) controlling seminal root number (Liu et al., 2013). Two previously reported QTL for kernel number per spike (*QKNS.caas-4BS*) and spike number per unit area (*QSN.caas-4BS*) also located near the interval targeted by three SNPs (*Affx-109674658, Affx-88444969*, and *Affx-88596529*) on chromosome 4B for seedling biomass traits (<0.1 Mb) (Li et al., 2018). Additionally, one SNP (*Affx*-*88431037*) on chromosome 4D for SDWN was 1.7 Mb from *Rht-D1* controlling plant height. The close locations of the loci for root and seedling biomass traits in the current analysis and those reported previously for plant height and grain yield are in line with the strong relationship between those traits (Bai et al., 2013; El Hassouni et al., 2018). Considering that the LD block in this population is about 4 Mb, it is likely that the loci for root and seedling biomass in the current analysis are the same as those for plant height and grain yield. However, further experiments are required to confirm the genetic relationship between these loci.

Because of the stability of the 11 SNPs under different water conditions, we investigated the potential candidate genes for these 11 SNPs. Some of the genes located within the 4-Mb LD block on each side of the 11 SNPs were known to be associated with plant growth and development based on previous literature (Table 2). For example, an *Arabidopsis* gene *AtOFP1* could suppress cell elongation and regulate cotyledon development in a postembryonic manner (Wang et al., 2007, 2011). In the current study, *TraesCS1B02G310200*, a wheat homologous gene of *AtOFP1*, is only about 95 kb from *Affx*-*109979441* on 1B controlling several seedling biomass traits and RDWN. Previous research found that a mutant of rolled and erect leaf 2 (*REL2*) gene is associated with the increased ability of rice leaves to capture light energy and exchange gas and thus increase the yield of rice (Yang et al., 2016). In this study, we found two homologous genes of the rice *REL2*, one is *TraesCS2B02G423500* near *Affx*-*109782056* on chromosome 2B, and the other is *TraesCS2D02G402400* near *Affx*-*109617080* on chromosome 2D (Supplementary Table 3). The expression of *OsTMK* is particularly high in regions undergoing cell division and elongation, and low in the non-growing region of the internode, implying that *OsTMK* regulates rice growth (Van Der Knaap et al., 1999; Hirose et al., 2007). *TraesCS4B02G048400*, a homologous gene of *OsTMK* was located 1 Mb away from *Affx*-*109674658* on chromosome 4B. Kurepa et al. (2009) showed that partial loss of function of the regulatory particle non-ATPase (RPN) subunits RPN10 and RPN12a caused a stronger defect in proteasome function and also resulted in cell enlargement and decreased cell proliferation in *Arabidopsis*. The gene *TraesCS4B02G049700* near the SNP *Affx*-*88444969* is homologous to the *Arabidopsis* gene *RPN12a* (Supplementary Table 3). Further experiments will be carried out to determine the functions of the above genes in root and seedling development.

## Data Availability Statement

The datasets presented in this study can be found in online repositories. The names of the repository/repositories and accession number(s) can be found in the article/Supplementary Material.

## Author Contributions

JM conceptualized and designed the research. IF, YG, XX, and JJ performed experiment. JM, IF, YG, and XX analyzed data and wrote the manuscript. JM, WZ, CX, and SD discussed the results and revised the manuscript. All authors have read and approved final manuscript.

## Conflict of Interest

The authors declare that the research was conducted in the absence of any commercial or financial relationships that could be construed as a potential conflict of interest.
